# Evolution of Biomimetic Approaches for Regenerative and Restorative Dentistry

**DOI:** 10.7759/cureus.33936

**Published:** 2023-01-18

**Authors:** Meghna Paryani, Priyanka R Bhojwani, Anuja Ikhar, Amit Reche, Priyanka Paul

**Affiliations:** 1 Department of Conservative Dentistry and Endodontics, Sharad Pawar Dental College and Hospital, Datta Meghe Institute of Medical Sciences, Wardha, IND; 2 Department of Public Health Dentistry, Sharad Pawar Dental College and Hospital, Datta Meghe Institute of Medical Sciences, Wardha, IND

**Keywords:** adhesive dentistry, restorative dentistry, revascularization, biomimetic microenvironment, regenerative endodontics

## Abstract

Biomimetics refers to human-made processes, substances, systems, or devices that imitate nature. The art and science of designing and building biomimetic apparatus are called biomimetics. This method can be widely used in dentistry to restore the structure and function of normal tooth structure. Traditional approaches to treating damaged and decayed teeth require more aggressive preparation to place a “strong,” stiff restoration. The emphasis was made on the strength of the restoration as well as its function and mechanical properties, despite several disadvantages like tooth fracture, making future treatment more difficult and invasive. This review paper will seek to provide a clear explanation of its scope, different fields of biomimetic dentistry, and materials used in biomimetics that improve the strength of the tooth.

## Introduction and background

Biomimetics studies the structure and function of the biological product, formed by biological mechanisms and processes required for synthesizing artificial materials that mimic natural tissues [1]. Otto Herbert Schmitt [2], a biophysicist/biomedical engineer, coined the term biomimetic in the 1950s [2] in his multidisciplinary study of the biological formation of a material that mimics life [1,2]. The principle of this material is the comprehension of teeth [3-5]. In dentistry, the principle followed is not just the formation of tooth structure but also the establishment of its function, stress-bearing, and esthetics [6-8]. The knowledge of biomimetics deals with the development of principles of nature in technological applications and devices, processes, and construction from biological to technical. Achieving a material that is formed innovatively at all the stages of abstraction was successful [9,10]. An increase in the durability of dental restorative material in dentistry is achieved by nanofiller materials [11,12].

The requirement for new kinds of bioinspired material leads to introducing biomimetic bond composition as an essential clinical and scientific task [13-15]. One of the most commonly used dental materials as filler and bond is calcium hydroxide having higher efficiency due to similar physicochemical properties with the inorganic constituents to dental hard tissues and bone. Hydroxyapatite-based materials have regenerated and replaced tissue to modify cement bonding [16-18]. Biomimetically restored tooth results in deformation and stress concentrations, eliminating sensitivity and postoperative pain and preserving vitality, as bacteria cannot invade and kill the pulp. A tooth's natural fracture resistance and flexibility are also enhanced when it is hydrated by the vital pulp [19-21].

The two significant properties on which attention to biomimetics is achieved are their property of regeneration of biological tissue and the property to restore the characteristics of the biological effect of these tissues [22]. However, both these properties have one aim to mimic the biological properties of the tooth by restoration. The modulus of elasticity of the material and its function should match the dental tissue (e.g., enamel, dentin, pulp, dentinoenamel junction) [23]. Cell-homing strategies for forming pulp cell-homing, pulp-capping agent regenerating dentin barrier, apexogenesis, apexification leading to root formation, and root-end fillings resulting in apical healing are all applications of biomimetic endodontic regeneration [24-26]. This article aims to review the various methods of biomimetics and materials that use restorative biomaterials to replace diseased or damaged dental tissues. The review also enlightens the findings of multiple studies regarding dental tissue properties and dental restorative materials properties. To restore the tooth's function, esthetics, and strength, regenerative endodontics has been presented as a way to replace decayed and undeveloped tooth structures with healthy pulp dentin tissue. In the conventional method, more tooth structure is removed, and hard material is substituted. However, it decreases the durability of the restoration as well as the tooth.

## Review

Methodology

**Figure 1:**
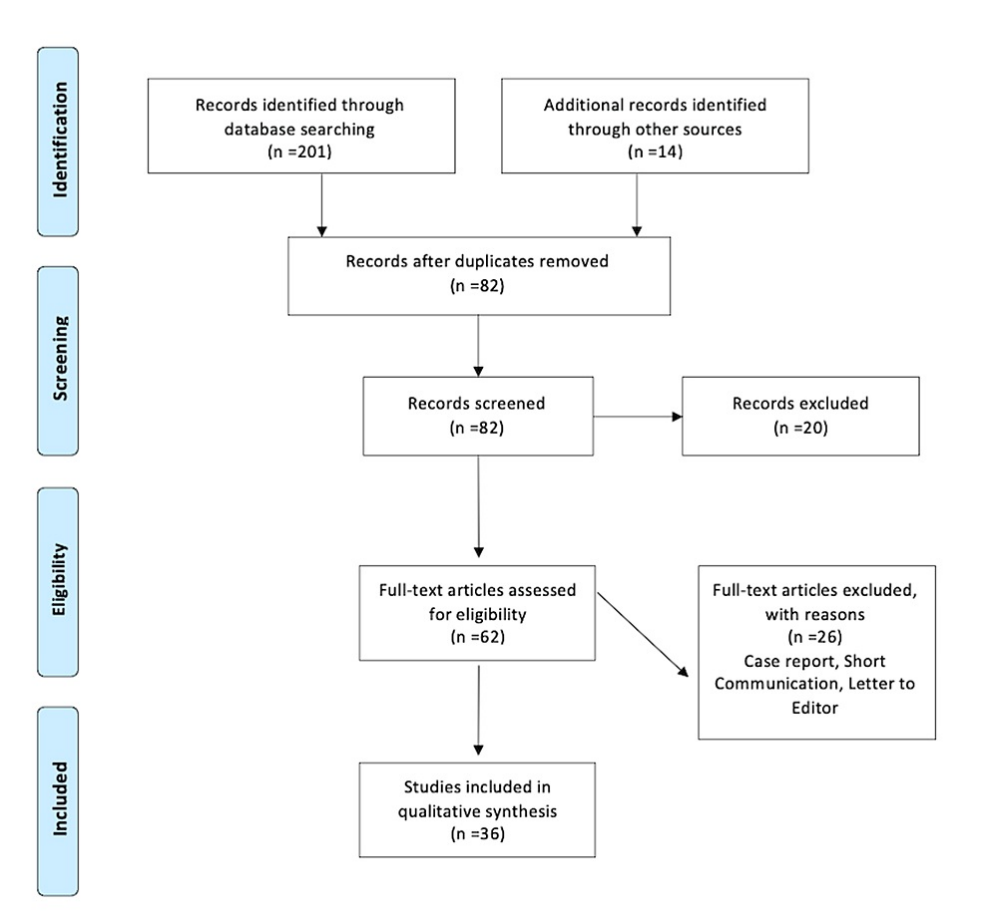
Flow diagram of literature review

This review's goal is to reembrace the knowledge regarding biomimetic material and also to focus on the future need for this material. It also brings the dentist close to biomimetics by giving knowledge, information, and an interest in further research. PubMed, Google Scholar, Embase, and Medline electronic databases were used for the search of the English-language literature, and the search terms were adhesive dentistry, restorative dentistry, revascularization, biomimetic microenvironment, and regenerative endodontics. The writers' personal knowledge and experience in the field supported the archiving of relevant papers. Articles that match the following criteria are included in this review: studies in English, studies from the last few decades, and studies devoted entirely to restorative materials. The research methodology by Preferred Reporting Items for Systematic Reviews and Meta-Analyses (PRISMA) is shown in Figure 1.

Biomimetic materials used in conservative dentistry

The material used to restore the function of the tooth should exhibit properties such as modulus of elasticity, tensile strength, and compressive strength for the replaced tooth structure [9]. In this article, the properties of hydroxyapatite, glass ionomer cement, calcium hydroxide, self-healing composites, mineral trioxide aggregate, DoxaDent, Ceramir, TheraCal, bioactive glass, Emdogain, and Ceramicrete are compared to those of the natural tooth.

Hydroxyapatite

Hydroxyapatite is a nonrestorable calcium phosphate material. It is composed similarly to bone and has an osteoconductive characteristic. Due to its poor mechanical properties, it is not employed in areas that sustain loads. It serves as a filler in composite resin and is used in bone grafting. For endodontic therapy, hydroxyapatite has been used for perforation repair, formation of the apical barrier, pulp capping, and periapical defect repair. When compared to calcium hydroxide-produced reparative dentin, Malik et al. claimed that tricalcium phosphate hydroxyapatite produces dentin mineralization that is more extensive and thicker [27].

Glass ionomer cement

Glass ionomer cement is regarded as a biomimetic material because it exhibits characteristics of dentin, such as adhesiveness to tooth structures and fluoride release. It is a restorative material that is bioactive and has a wide range of uses, such as bonding, lining, luting, sealing, or restoring teeth. It has a coefficient of thermal expansion identical to that of a natural tooth [22,23,28].

Calcium hydroxide

Calcium hydroxide was introduced by Hermann in dentistry. It contains calcium ions and hydroxyl ions. Hydroxyl ions neutralize the acid produced and maintain the pH for the activity of the pyrophosphatase leading to the increasing level of calcium-dependent pyrophosphatase, which decreases the levels of inhibitory pyrophosphate and causes mineralization. It is used as a cavity liner, as an interim root canal dressing to induce hard tissue formation, in the treatment of root fracture and root resorption, and as a permanent root canal sealer. It has antibacterial properties due to the alkaline pH, and it may aid in dissolving necrotic tissue remnants, bacteria, and their byproducts. It also has the ability to induce tertiary dentin formation [28].

Self-healing composites

Self-healing composites include polyurea-formaldehyde (PUF) or silica microcapsules. Silica microcapsules use water or polyacid as a healing agent. They are fabricated to repair cracks and damages, if there will be any, to restrict failure and extend the longevity of structures. In case of composite resin, if cracks are seen, the microcapsules are destroyed near the crack and the resin is released. When the catalyst placed in the epoxy composite reacts with the resin to fill the crack, the resin polymerizes and the crack is repaired [29].

Mineral trioxide aggregate

Mineral trioxide aggregate (MTA) is a hydrophilic substance made of calcium silicate that was created by Torabinejad in 1990. With a pH range of 10 to 12, it crystallizes calcium hydroxide and crystals resembling hydroxyapatite when exposed to phosphate-containing solutions. Gypsum, tetra-calcium aluminoferrite, tricalcium aluminate, dicalcium silicate, and tricalcium silicate are all components of MTA. This is a preferred substance for pulp capping, root-end filling in apicoectomy procedures, vital pulp therapy, apexogenesis, and apexification. It results in cement development, dentinal bridge construction, and periodontal ligament attachment. When implanted, it stimulates the growth and development of odontoblast-like cells, which result in the production of a collagen matrix. The matrix is thus first mineralized by osteodentin and later by the development of tertiary dentin. It shows good adhesion to dentin. When employed in vital pulp therapy, it has low solubility, and no tunnel defects are seen as compared to calcium hydroxide [27,28,30].

DoxaDent

The calcium and aluminum present in this cement, which was first developed in 2000, react with water that contains salts of lithium to produce gibbsite and katoite. It is inorganic and non-metallic in nature. It is available as a liquid powder component. It is a tough substance with little wear resistance. It is equally potent as glass ionomer cement. Alumina, zirconium dioxide, calcium dioxide, water, and other alkaline oxides make up its constituent parts. It is employed as a long-lasting reparative material [27,28].

Ceramir

Ceramir is used for long-term cementation and contains calcium aluminate. The calcium released reacts with alkaline pH to rebuild dentin and enamel of all zirconia, inlay, gold, and fixed partial dentures. It reacts favorably with the inorganic phosphate in saliva to form hydroxyapatite and exhibits good gingival reaction when used as a luting agent [28].

TheraCal

TheraCal is made of light-cured silicate resin that has been modified with resin. It serves as a protective layer underneath base materials including cement, amalgam, and composite. Compared to Dycal and MTA, TheraCal has low calcium solubility and high calcium release [27,28].

Bioactive glass

They have the ability to react in liquid or water. The formation of silica gel polycondensed coating on glass bulk serves as a template for the development of calcium phosphate due to its characteristics and high biocompatibility. Bioactive glasses have also been used in bioregeneration. These can be utilized for implant coating, dentin hypersensitivity treatment, and bone grafting [31,32].

Emdogain

Emdogain is made from enamel matrix protein from the tooth germ of swine and propylene glycol alginate as a matrix. Hertwig epithelial root sheath secretes an enamel matrix-derived protein that induces the formation of periodontal tissue. Emdogain imitates these tooth-developmental mechanisms. Ameloblastin, enamelin, growth factor, tuftelin, and bone morphogenic protein are examples of non-collagen proteins that are also present in Emdogain. It has been used in the treatment of vital pulp therapy and pulpotomy because it causes reparative dentin formation. It is used to reduce external root resorption in replantation situations [27,31].

Ceramicrete

Ceramicrete is a radiopaque filler made of cerium oxide and powdered hydroxyapatite and is a new-generation calcium-based substance. It releases calcium and phosphate ions when setting and is radio-opaque and biocompatible. When utilized as a root-end filling material, it has a greater sealing capacity when compared to Pro-Root MTA. The surface of the Ceramicrete material forms hydroxyapatite or dicalcium phosphate dihydrate (DPCD) when it is immersed in a phosphate-containing fluid (PCF). The setting time is 2.5 hours. It has an initial pH of 2.2, which increases with time.

Discussion

Biomimetics material has been attributed to innumerable research, and this research has contributed immensely to the understanding and development of more and more materials (Table 1).

**Table 1:** Studies included in this review BisGMA: Bisphenol A-glycidyl methacrylate; cHAp; Calcium hydroxyapatite.

S. No.	Author	Title of the study	Inference
1	Kottoor [1]	Biomimetic endodontics: barriers and strategies	The practice of endodontics has grown in the past few decades. The future of endodontics will involve the use of materials that will replace lost tooth structures.
2	Harkness [2]	An idea man (Otto Herbert Schmitt)	The concept of a biomimetic approach to science and engineering is an idea rather than a gadget.
3	Magne [3]	Composite resins and bonded porcelain: the post-amalgam era?	The study focuses on understanding the intact tooth and the principle of biomimetic material and indications for a porcelain restoration.
4	Magne [4]	Manual for Posterior Esthetic Restorations: Esthetic and Biomimetic Restorative Dentistry	The study focuses on understanding the principles of biomimetics and indications for posterior aesthetic restoration.
5	Bottacchiari [5]	Composite Inlays and Onlays: Structural, Periodontal, and Endodontic Aspects	While composite offers both the chance for correction prior to cementation and long-term reparability, inlays and onlays ensure the preservation of sound tooth structure without compromising the tooth's mechanical and physical qualities.
6	Gebeshuber and Drack [6]	An attempt to reveal synergies between biology and mechanical engineering	Most of the biomimetic applications available today focus on construction. These basic principles comprise integration instead of additive construction, optimization of the whole instead of maximization of a single component feature, multifunctionality instead of monofunctionality, energy efficiency, and development via trial-and-error processes.
7	Torchilin [7]	Multifunctional pharmaceutical nanocarriers: development of the concept	More growth is expected in the field of biomimetics.
8	Bar-Cohen [8]	Biomimetics: biologically inspired technologies	Biomimetics is the wealth of inventions in nature as an inspiration for human innovation.
9	Neville [9]	Special issue on biomimetics in engineering	Special issues on biomimetic materials.
10	Nachtigall [10]	Grundlagen und Beispiele für Ingenieure und Naturwissenschaftler	Creative formation in the biomimetic materials by various stages of abstractions and modifications and successful construction of a “new invention” than a blueprint of nature.
11	Alhenaki et al. [11]	Dentin bond integrity of filled and unfilled resin adhesive enhanced with silica nanoparticles-an SEM, EDX, micro-Raman, FTIR, and micro-tensile bond strength study	The study was to assess and synthesize filled and unfilled dentin adhesive polymers.
12	Furtos et al. [12]	Nano-forsterite biocomposites for medical applications: mechanical properties and bioactivity	The aim of the study was to obtain and investigate nano-forsterite and nano-forsterite biocomposites for biomedical applications.
13	Goswami [13]	Biomimetic dentistry	Although much more scientific development and technical research are required, a good prognosis, high biocompatibility, and excellent success rate have been observed with the regeneration of lost dental tissues. There is a lot of scope in research of biomimetics in dentistry in India in the future because of their expected ability to regenerate dental tissues, i.e. enamel, dentin, pulp, and cementum.
14	Lagazzo et al. [14]	Molecular level interactions in brushite amino acids composites	The interaction between aminoacid and brushite-based bone cement has been studied by various techniques.
15	Kwak et al. [15]	Biomimetic enamel regeneration mediated by leucine-rich amelogenin peptide	The novel emphasizes on biomimetic approach to the regeneration of human enamel.
16	Provenzi et al. [16]	Interface evaluation of experimental dental adhesives with nanostructured hydroxyapatite incorporation	This study observed how resin and nanostructured hydroxyapatite penetrate the hybrid layer.
17	Furtos et al. [17]	New composite bone cement based on hydroxyapatite and nanosilver	The time of silver release increased depending on the silver content in the cement.
18	Dorozhkin [18]	Calcium orthophosphate-containing biocomposites and hybrid biomaterials for biomedical applications	CaPO_4_-based biocomposites and hybrid biomaterials need a good research approach in the future.
19	Brannstrom [19]	Dentin and pulp in restorative dentistry	The natural fracture resistance and flexibility of a tooth are also enhanced when it is hydrated by the vital pulp.
20	Brännström [20]	The hydrodynamic theory of dentinal pain: sensation in preparations, caries, and the dentinal crack syndrome	Bacterial products show an inflammatory response resulting in hyperalgesia. The breakage of the fractured cusp by chewing reduces the sensitivity.
21	Alleman and Magne [21]	A systematic approach to deep caries removal endpoints: the peripheral seal concept in adhesive dentistry	Complete caries removal without vital pulp exposure is achieved using caries-detecting dye and DIAGNOdent laser fluorescence technologies.
22	Srinivasan and Chitra [22]	Emerging trends in oral health profession: the biomimetic-a review	Reproduction in science is achieved by knowing the correct properties of natural tissue.
23	Chaitra et al. [23]	Biomimetics in dentistry-a review	Biocompatible restorative materials are used to replace the damaged or missing tooth structure, whereas biomimetic materials are expected to be used in the future to restore the dental tissues that are missing.
24	Bottino et al. [24]	Bioactive nanofibrous scaffolds for regenerative endodontics	A polymer-based drug delivery system such as antibiotic-containing electrospun scaffolds is used for regenerative endodontics.
25	Murray et al. [25]	Regenerative endodontics: a review of current status and a call for action	Regenerative endodontics is referred to as inevitable therapy and expects more development.
26	Yang et al. [26]	Pulp regeneration: current approaches and future challenges	In some experimental and clinical studies, vascular tissue resembling pulp is formed but they do not tell much about the function of the formed tissues.
27	Malik et al. [27]	Bio-mimetic materials in restorative dentistry: a review	This study evaluates various biomimetic materials that mimic the natural tooth structure.
28	Sonarkar and Purba [28]	Bioactive materials in conservative dentistry	Bioactive materials can be considered a boon to dentistry because of their regeneration potential.
29	Gurumurthy et al. [29]	Self-healing materials: a new era in material technology: a review	Coalescence and formation of microcracks will reduce the lifetime and failure of the materials. Microcracks are the problem during their use in structural applications.
30	Jefferies [30]	Bioactive and biomimetic restorative materials: a comprehensive review. Part I	Biodentine could be used as a dentin substitute that can be used under a composite for posterior restorations.
31	Sharma et al. [31]	Modern approaches to use bioactive materials and molecules in medical and dental treatments	Bioactive materials and molecules have antibacterial, antifungal, and antitumor importance with varying spectra of activities.
32	Bahrololoom et al. [32]	Bioactive glasses in dentistry: a review	The bioactive glass’s ability to bond with soft tissue and hard tissue and induce bone growth is commendable. This is observed as the generation of hydroxyapatite on the surface.
33	Dodwad and Kukreja [33]	Biomimetics-the new pathway for regenerating tissue	The major goal of regenerative therapy has been to reconstruct the alveolar bone-supporting tooth. When designed appropriately, the material would be able to respond in a more effective and controlled manner and will provide an innovative new arena in the research and development of biomaterials.
34	Farhana et al. [34]	Biomimetic materials: a realm in the field of restorative dentistry and endodontics: a review	Biomimetic materials will help the future dentist to provide a treatment that gives better outcomes to the patient by not only regenerating enamel, dentin, and cementum but also pulp tissue. A thorough tissue engineering has been employed to completely replace the lost tooth structures that mimic the biological properties of natural dental tissues. A revolution to the future of dentistry is biomimetics and its materials.
35	Zafar et al. [35]	Biomimetic aspects of restorative dentistry biomaterials	The researchers of this study look forward to the availability of complete regeneration of dental tissues that mimic the mineralized nano-structural, biological, and mechanical properties of natural tooth tissues.
36	Seredin et al. [36]	The molecular and mechanical characteristics of biomimetic composite dental materials composed of nanocrystalline hydroxyapatite and light-cured adhesive	Using light-cured BisGMA adhesive and nano-cHAp, its characteristics on enamel and dentin were concluded as a calcium source. This result has an effect on the success and application of the developed biomimetic adhesives for tooth restoration.

Dodwad et al. [33] reported that the major goal of regenerative therapy has been to reconstruct alveolar bone-supporting teeth. Limited success is seen in oral and craniofacial tissue engineering with the use of bone substitutes, cell occlusive barrier membranes, and autogenous block grafting techniques. Tooth support lost by periodontal disease or trauma is restored using signal molecules, i.e., growth factors, although all these and other methods of reconstruction do not completely mimic natural tissue. Though designed appropriately, the material would be able to respond in a more predictable, effective, and controlled manner to provide an innovative new arena in the research and development for biomaterials [33].

Farhana et al. [34] investigated biomimetics, which is the study of regenerative materials used for the regeneration of a structure's biological function and material. Biomimetics aims to synthesize a material that uses artificial mechanisms and mimics natural dental tissues. The material fabricated by biomimetics based on biological processes is called biomimetic material. The order of treatment today is the replacement of lost or diseased tooth structure with a restorative material. However, it has certain disadvantages. Although regeneration of the tissues rather than replacement overcomes these disadvantages, these biomimetic materials will help the future dentist to provide a treatment that gives better outcomes to the patient by not only regenerating enamel, dentin, and cementum but also pulp tissue. A thorough tissue engineering has been employed to completely replace the lost tooth structures that mimic the biological properties of natural dental tissues. A revolution to the future of dentistry is biomimetics and its materials [34]. Sonarkar et al. [28] found that bioactive materials can be considered a boon to dentistry because of their regeneration potential. Chaitra et al. [23] currently practiced to replace damaged or missing tooth structures with biocompatible restorative materials; however, each of these methods has its own limitations and cons. Instead of replacing the missing tooth structure, regeneration will ensure a more favorable outcome and a higher success rate. Therefore, biomimetic materials would be used in dentistry in the future to successfully restore missing enamel, dentin, cementum, and even pulp tissue.

Goswami [13] acquitted that biomimetics has proven to be very useful. Although much more developed scientific and research techniques are required, a good prognosis, high biocompatibility, and excellent success rate have been observed with the regeneration of lost dental tissues. In India, there is a lot of scope for research of biomimetics in dentistry. According to Zafar et al. [35], many types of research have been conducted with the aim of developing biomimetic materials, either by changing some components in the existing biomimetic material or by completely developing a new biomimetic material. Different technologies such as nanotechnology, fabrication methods, and functioning of biomaterials have been employed for the development of biomimetics. In the past 10 years, biomimetics has gathered a lot of attention due to their appreciable simulating properties of the natural tissues, but due to the extremely complex properties of natural tissues, the research of biomimetics is still considered to be in the first stage and has a lot more scope of development. In the past decades, great development in a multifaceted fast-emerging field is seen by experiencing exponential growth in biomimetic tissue engineering. The researchers of this study look forward to the availability of complete regeneration of dental tissues that mimic the mineralized nano-structural, biological, and mechanical properties of natural tooth tissues [35].

Seredin et al. [36] studied the molecular and mechanical characteristics of biomimetic composite dental materials composed of nanocrystalline hydroxyapatite and light-cured adhesive using light-cured bisphenol A-glycidyl methacrylate (BisGMA) adhesive and nano-calcium hydroxyapatite (nano-cHAp); its characteristics were concluded as a calcium source on enamel and dentin. Nano-cHAp filler with the adhesive mixture showed a change in the chemical bond via FTIR spectroscopy. Nanofiller of specified concentration Vickers hardness (VH) values were increased. The degree of conversion was observed in light-cured BisGMA/nano-cHAp adhesive. This result has an effect on the success and application of the developed biomimetic adhesives for tooth restoration.

## Conclusions

The notion of biomimetics in dentistry has a lot of importance, and many studies have been conducted, either to modify the existing material or to develop new material. It is more likely to be successful, have a better prognosis, and have superior biocompatibility if the lost dental tissue is replaced rather than mild replacement with dental materials. Dentin, enamel, cementum, and pulp that have been lost could be successfully replaced through biomimetic dentistry, opening a new era of dentistry. The last few decades have seen tremendous growth in the field of dentistry. But each procedure has its own drawbacks and limitation due to the complex natural tooth structure. Therefore, the utilization of such biomimetic materials that could successfully restore the destroyed enamel, dentine, dentinoenamel junction, cementum, and even the pulp tissue will be required in the future of dentistry. The development of a substitute that restores or mimics the natural dental tissue is in progress. Moreover, the role of various biomimetic molecules and materials requires further study.
